# p53-dependent chromatin relaxation is required for DNA double-strand break repair: p53 directly increases DSB damage repair efficiency

**DOI:** 10.3724/abbs.2025008

**Published:** 2025-02-25

**Authors:** Hongyu Chen, Jin Shan, Wenjing Qi, Lili Chen, Xianlu Zeng

**Affiliations:** 1 The Key Laboratory of Molecular Epigenetics of Ministry of Education Changchun 130024 China; 2 Institute of Neuroscience Shanghai 200031 China; 3 Institute of Systems and Physical Biology Shenzhen 518107 China; 4 Department of Bioscience Changchun Normal University Changchun 130032 China; 5 College of Life Science Liaoning University Shenyang 110036 China

**Keywords:** DNA damage repair, DNA double-strand breaks (DSBs), p53, chromatin relaxation

## Abstract

The tumor suppressor p53, an indispensable nuclear transcription factor, plays a central role in orchestrating cellular responses when DNA damage occurs. In this study, we demonstrate that in the initial phases of DNA double-strand break (DSB) repair, p53 is rapidly recruited to sites of damage and the surrounding chromatin, where it enhances DSB repair efficiency. This enhancement occurs through the modulation of chromatin dynamics and the promotion of a more relaxed chromatin configuration, a process influenced by p53 in response to DSB-inducing factors such as etoposide, ultraviolet radiation, and nucleases. These results underscore the pivotal function of p53 as a rapid responder to DSBs, delineating a significant departure from its traditionally recognized role as a downstream transcriptional regulator in DNA damage repair processes. This study emphasizes that the direct engagement of p53 in DNA repair through chromatin structure regulation extends beyond its established involvement in UV irradiation-induced nucleotide excision repair (NER), demonstrating analogous mechanistic attributes in the context of DSB repair. This newly illuminated perspective enhances our understanding of the multifaceted roles of p53 in genome stability and integrity.

## Introduction

DNA, while being a comparatively stable organic molecule, is perpetually exposed to a multitude of endogenous and exogenous damaging agents. In response to this relentless challenge, cells have developed an intricate array of biochemical pathways designed to mitigate such threats [ 1, 2] . This advanced regulatory network is collectively referred to as the DNA damage response (DDR), which orchestrates a variety of critical biological outcomes, such as cell cycle arrest, DNA repair, apoptosis, and cellular senescence [3]. The components of the DDR are strategically classified into sensors, signal transducers, and effectors to address cellular challenges efficiently. A DNA double-strand break (DSB) represents a significant challenge for cells, with accurate repair being crucial for maintaining cellular survival and preventing oncogenic translocations [4]. A paradigmatic example of the organizational structure of the DDR is the recruitment of the MRN (MRE11-RAD50-NBS1) complex to DSB sites. The MRN complex is instrumental in recruiting and activating the ataxia telangiectasia mutated (ATM) kinase, which is pivotal in the DDR. In response to DNA damage, ATM transitions from an inactive dimer to an active monomer through autophosphorylation at key serine residues, such as S1981 [5]. Once activated at a damage site, ATM phosphorylates H2AX at S139, producing γ-H2AX, which in turn recruits MDC1, establishing a feedback loop that enhances ATM signaling by attracting more MRN complexes and ATM molecules [6]. This activation cascade also involves other critical factors, such as 53BP1, BRCA1, and the ubiquitin ligases RNF8 and RNF168 [ 7, 8] . Among the most prolific ATM substrates, the tumor suppressor p53 is at the top [9].

DSB repair involves two major pathways: homologous recombination (HR) and nonhomologous end-joining (NHEJ). The key difference between HR and NHEJ is that HR is recruited exclusively during the S and G2 phases of the cell cycle; in contrast, NHEJ can be activated during all cell cycle phases [ 10, 11] . HR is an error-free repair mechanism that uses a homologous template to accurately reassemble damaged DNA strands. Key proteins in HR include the MRN complex, BRCA1/BRCA2, ATM, and ATR, which are essential for initiating and regulating this process [11]. Conversely, NHEJ lacks a homologous template, directly rejoining DNA ends and thus being inherently error prone. Critical NHEJ factors include the Ku70/Ku80 complex, DNA-dependent protein kinases, and XRCC4, which are pivotal in this repair pathway [10]. The eukaryotic DSB response takes place on chromatin near the break site; thus, the basic organization of eukaryotic chromatin needs to be considered. Chromatin relaxation is considered a fundamental pathway in the DNA damage response [12].

P53 was initially found to be associated with the T antigen of simian virus 40 in rodent cells that had undergone transformation [13], and its function as a tumor suppressor was first acknowledged in 1989 [ 14, 15] . Under nonstressful conditions, the E3 ubiquitin ligase MDM2 maintains minimal p53 levels [ 16, 17] . However, with the activation of cellular stress, p53 becomes phosphorylated, which makes it resistant to MDM2, resulting in an increase in p53 in cells [18]. p53 plays a pivotal role in the response to cellular stress and can control the transcription of hundreds of genes involved in several biological functions, such as DNA damage repair, cell cycle arrest, apoptosis, and senescence [ 19, 20] . In the context of DNA damage repair, p53 is viewed as an essential transcription factor that selectively triggers the activation or inhibition of genes within particular gene expression programs to guide the destiny of cells. However, recent studies have pinpointed p53 as an element of the replication apparatus, often referred to as the replisome, which plays a direct role in protecting against stress caused by replication [ 21, 22] . Similarly, the nontranscrip tional roles of p53 in affecting cellular repair have been demonstrated by the protein complexes with which p53 interacts [ 23, 24] . These findings indicate that p53 may play a previously unknown role in sensing and mitigating the cellular stress and DNA damage that undermine genome stability. This might occur much earlier than what is commonly expected, in which p53 plays a role as a major transcriptional effector in the DDR signaling cascade. The role of p53 could be distinct from its established function in the DDR signaling cascade. These findings support this view, revealing the effects of p53 deficiency on genome stability and tumor suppression. Importantly, these effects cannot be completely attributed to the systematic perturbation of the downstream DDR and the repair of transcriptional targets of p53 [ 25, 26] . This prompted us to reexamine the function of the p53 response to DSB repair.

In this study, we found that there is a positive correlation between the accumulation of p53 protein and the severity of DSB damage. Compared with that in wild-type cells, the efficiency of DSB repair is significantly lower in cells with impaired expression of p53. Under DSB damage, p53 rapidly enters the cell nucleus and binds to chromatin both near and far from the damage site. In the early stages of DSB repair, p53 can increase repair efficiency by directly regulating chromatin structure and promoting chromatin relaxation, thus directly contributing to improved DSB repair efficiency. Importantly, p53 can directly engage in DSB damage repair, irrespective of its transcriptional activity.

## Materials and Methods

### Antibody and reagents

Anti-p53 (sc-126), anti-Cyclin A (sc-751) and anti-PARP1 (sc-74470) were obtained from Santa Cruz Biotechnology (Dallas, USA); anti-γH2AX (Ser319) (#9718S) were obtained from Cell Signaling Technology (Boston, USA); anti-β-actin (66009-1-Ig), anti-MCM6 (13347-2-AP), anti-PCNA (60097-I-Ig), anti-H3(17168-I-AP) and anti-H2AX (PA1-41004) were obtained from Invitrogen (Shanghai, China); anti-GAPDH (HT801) was obtained from TransGen Biotech (Beijing, China); anti-H2A (GTX129418) was obtained from GeneTex (Shanghai, China); anti-H2B (BS2568) was obtained from Bioworld Technology (Nanjing, China); nuclear extract from HeLa cells (KI-140) was obtained from BIOMOL (Shanghai, China); and Etoposide (Eto), Camptothecin (CPT), Puromycin and RNase A were purchased from Sigma-Aldrich (Merck KGaA Darmstadt, Germany); Propidium iodide (PI) solution was purchased from DingGuo (Beijing, China); DMEM and RPMI 1640 medium were obtained from Gibco (Grand Island, USA); TRIzol reagent, PrimeScriptTM RT reagent Kit and SYBR® Premix Ex TaqTM were purchased from TaKaRa (Shiga, Japan); Lipofectamine 2000 reagent were purchased from Invitrogen (Carlsbad, USA); ECL Plus western blot detection reagents was obtained from Super ECL Str (US Everbright Inc, New York, USA).

### Cell culture and treatment

Human HCT116 ^WT^ and HCT116 ^p53–/–^ cells were kindly provided by Dr. Yong Cai (Jilin University, Changchun, China) and were maintained in RPMI 1640 medium supplemented with 10% fetal bovine serum (FBS). 293T cells (American Type Culture Collection, Manassas, USA), U2OS cells (American Type Culture Collection), U2OS-shCTR cells, U2OS-shp53 cells and Asi-ER-U2OS cells were cultured in DMEM supplemented with 10% FBS. For DSB damage induction, the cells were incubated with the indicated concentrations of Eto for 20 min, after which the medium was replaced by fresh medium to repair the damage.

The plasmids encoding the GFP-RNF8 ^WT^ and GFP-RNF8 ^C403S^ plasmids were kindly provided by Dr. Nico P. Dantuma [27]. The pBABE HA-ER-AsiSI plasmid was generously provided by Gaelle Legube. The GFP-p53 plasmid was constructed by cloning the full-length cDNA of p53 into a PEGFP-C1 vector (Addgene, Watertown, USA). The GST-p53 plasmid was constructed by cloning the full-length cDNA of p53 into a pGEX-4T-2 vector (Addgene). To stably knock down p53 in U2OS cells, the p53 shRNA and shCTR sequences were synthesized and cloned and inserted into the pLKO.1-TRC Cloning Vector (Addgene). In addition, the psPAX2 and pMD2.G plasmids were purchased from Addgene. All primers and shRNAs used for plasmid construction are presented in Table 1.

**Table:** **Table 1** Sequence of shRNAs used for constructing shRNA plasmids

Name	Sequence (5′→3′)
TRC-PLKO.1-shCTR	ACGCTGAGTACTTCGAAATGT
TRC-PLKO.1-shp53#1	GAAATTTGCGTGTGGAGTA
TRC-PLKO.1-shp53#2	GCGCACAGAGGAAGAGAAT
TRC-PLKO.1-shp53#3	GCACAGAGGAAGAGAATCT

### Plasmid transfection and lentiviral infection

Most of the transfection experiments were performed according to the manufacturer’s instructions for Lipofecta-mine™ 2000. For the pBABE HA-ER-AsiSI plasmid, the transfections were performed in 60-mm dishes containing 70% confluent cells with 4 μg of DNA per dish. AsiSI-ER-U2OS cells stably transfected with the pBABE HA-AsiSI-ER plasmid were selected with 1 mg/mL puromycin. Isolated individual Asi-U2OS clones were further validated by western blot analysis. For lentiviral transduction, the TRC-PLKO.1-shCTR, TRC-PLKO.1-shp53#1, TRC-PLKO.1-shp53#2 and TRC-PLKO.1-shp53#3 plasmids were separately transfected into HEK-293T cells, together with the packaging plasmid psPAX2 and the envelope plasmid pMD2. G by using the Lipofectamine 2000 reagent. The lentiviral supernatants were harvested at 48 h, 72 h, and 96 h after transfection, after which the virus was concentrated, and the cells were injected. The infected cells were selected in puromycin-containing medium for 3 days after infection.

### Protein expression and GST pull-down assay

*Escherichia coli* strain BL-21 (DE3) was transformed with the indicated plasmids and cultured overnight. GST fusion protein expression was induced with isopropyl β-D-thiogalactoside (IPTG). The cells were harvested, lysed in lysis buffer (20 mM HEPES, pH 7.5, 120 mM NaCl, 10% glycerol, 2 mM EDTA, 1 mg/mL lysozyme, 1 mM PMSF, 10 μg/mL each aprotinin and leupeptin) and homogenized via sonication. After centrifugation, the GST-fusion proteins in the supernatant were purified with glutathione-Sepharose 4B beads (Amersham Pharmacia Biotech, Uppsala, Sweden) according to the manufacturer’s instructions. For the GST pull-down assay, the HeLa nuclear extract was incubated with 10 μL of beads coated with GST or GST-p53 fusion proteins for 3 h. The beads were collected by centrifugation and washed with ice-cold lysis buffer. After boiling in Laemmli sample buffer, the coimmunoprecipitated proteins were detected by western blot analysis.

### Microscopic imaging

For immunofluorescence (IF) staining, cells grown on glass coverslips were fixed with 10% (w/v) formaldehyde in PBS for 10 min and then permeabilized with 0.5% (v/v) Triton X-100 for 5 min. After permeabilization, the cells were washed and blocked in 10% FBS for 30 min. The cells were incubated with the primary antibody, washed and stained with a secondary antibody. For laser microirradiation, U2OS cells were grown on coverslips and incubated with Hoechst 33342 (2 μg/mL) for 5 min. Then, the cells were irradiated with a pulsed nitrogen laser (50 Hz, 405 nm) at 85% output power for 10 s, and pictures were taken at the indicated time points. All images were captured via a FluoView FV1000 confocal microscope (Olympus, Shenyang, China).

### Neutral cell comet assay

The neutral comet assay was performed via the Comet Assay Kit from Trevigen (Gaithersburg, USA) following the manufacturer’s instructions. Images were captured via a fluorescence microscope (ECLIPSE, 80i, Nikon, Japan). The tail moment was tested via Sciencetech, London, Canada.

### Chromatin isolation

A total of 4×10 ^6^ cells were washed with PBS. Then, 10% of the cell pellets were lysed in RIPA buffer to produce a whole-cell lysate control; the remaining portion was resuspended in 200 mL of solution A (10 mM HEPES, pH 7.9, 10 mM KCl, 1.5 mM MgCl _2_, 0.34 M sucrose, 10% glycerol, 1 mM DTT, 10 mM NaF, 1 mM Na _2_VO _3_ and protease inhibitors). Triton X-100 was added to a final concentration of 0.1%, and the cells were incubated for 10 min on ice. Cytoplasmic extracted proteins (CEs) were separated from the nuclei via low-speed centrifugation (4 min at 1300 *g*, 4°C). The isolated nuclei were washed once with solution A and then lysed in 200 mL of solution B (3 mM EDTA, 0.2 mM EGTA, 1 mM DTT and protease inhibitors) for 30 min. Insoluble chromatin was collected by centrifugation (4 min at 1700 *g*, 4°C), and the nucleolus-extracted protein (NE) was separated. Insoluble chromatin (Chr) was washed once with solution B and centrifuged again at high speed (1 min at 10,000 *g*). The final chromatin pellet was resuspended in 200 μL of Laemmli buffer and sonicated for 15 s. Chromatin was digested by resuspending in solution A containing 1 mM CaCl _2_ and 50 units of micrococcal nuclease (MNase; N3755, Sigma Aldrich,St. Louis, USA ) and incubating at 37°C for 1 min, after which the nuclease reaction was halted by the addition of 1 mM EGTA [28]. The lysates were analyzed by western blot analysis.

### Micrococcal nuclease (MNase) susceptibility assay

MNase assays were performed as described previously [29]. Briefly, cultured cells were harvested, centrifuged, and resuspended in ice-cold NP-40 lysis buffer (10 mM Tris-HCl, pH 7.4, 10 mM NaCl, 3 mM MgCl _2_, 0.5% NP-40, 0.15 mM spermine, and 0.5 mM spermidine) for 5 min. The resulting nuclei were pelleted and resuspended in MNase digestion buffer (10 mM Tris-HCl, pH 7.4, 15 mM NaCl, 60 mM KCl, 0.15 mM spermine, and 0.5 mM spermidine) containing 1 mM CaCl _2_. An undigested fraction of chromatin was stored at –20°C. The remaining chromatin was subjected to digestion with 5 U of MNase for 5 min at room temperature. The reaction was stopped by the addition of MNase stop buffer (15 mM EDTA and 2.3 mM EGTA). The digested samples were then treated with 60 μL of 10% (v/v) SDS, 10 mg/mL proteinase K and 10 μL of 10 mg/mL RNase for 15 min at 37°C. DNA was extracted using Tris-saturated phenol and chloroform. Finally, the DNA was ethanol precipitated and resuspended in Tris-EDTA (TE) buffer. The MNase-treated nucleosomal DNA was resolved on 1% agarose gels, visualized with ethidium bromide, and photographed with an Eagle Eye apparatus (Speed Light/BT Sciencetech-LT1000; Sciencetech, London, Canada). The cells were harvested, washed twice with ice-cold PBS and centrifuged at 200 *g*. The cell pellets were completely resuspended in 500 μL of buffer A (20 mM HEPES, pH 7.9, 0.5 mM DTT, 1 mM PMSF, 1.5 mM MgCl _2_, and 0.1% Triton) containing 0.15 M NaCl to remove the cells.

### Chromatin immunoprecipitation (ChIP) assay

The ChIP assay was performed as described previously [30], with slight modifications. Briefly, formaldehyde was added to the medium at a final concentration of 1% for 10 min, and the reaction was halted by the addition of 0.125 M glycine. The cells were subsequently washed and harvested by scraping. Pelleted cells were incubated in lysis buffer (50 mM Tris-HCl, pH 8.1, 10 mM EDTA, 1% SDS) and sonicated. The samples were diluted in dilution buffer (16.7 mM Tris-HCl, pH 8.1, 0.01% SDS, 1.1% Triton X-100, 1.2 mM EDTA, 67 mM NaCl) and precleared with blocked protein A/G beads (Sigma-Aldrich). Precleared samples were incubated with the indicated antibodies or isotype IgG overnight at 4°C. The immune complexes were then recovered by incubating the samples with blocked beads and were eluted twice by incubation in elution buffer (1% SDS, 100 mM NaHCO _3_) for 15 min. Crosslinking was reversed by addition of 5 M NaCl and RNase A to the samples and incubation overnight at 62°C. After proteinase K treatment, the immunoprecipitated and input DNA were purified with phenol/chloroform, precipitated and analyzed in duplicate via real-time qPCR performed on a StepOnePlus Real-Time PCR System (Applied Biosystems, Foster City, USA) with SYBR Green qPCR SuperMix (Invitrogen) according to the manufacturer’s instructions. We used primers located in close proximity to (3.7 kb) and distal to (2 Mb) the AsiSI site on chromosome 22 at position 19180307. All samples were calibrated for amplification from input DNA. Three biological replicates were performed. The primer sequences are presented in Table 2.

**Table:** **Table 2** Sequences of primers used for qRT-PCR analysis

Name	Forward primer (5′→3′)	Reverse primer (5′→3′)
*p21*	CCCGTGAGCGATGGAACT	CGAGGCACAAGGGTACAAGA
*BAX*	ACCAAGAAGCTGAGCGAGTGT	ACAAACATGGTCACGGTCTGC
*β-Actin*	CTCCATCCTGGCCTCGCTGT	GCTGTCACCTTCACCGTTCC
*GAPDH*	AACGGATTTGGTCGTATTG	GGAAGATGGTGATGGGATT
chr22:19180307-dist	CCCATCTCAACCTCCACACT	CTTGTCCAGATTCGCTGTGA
chr22:19180307-prox	CCTTCTTTCCCAGTGGTTCA	GTGGTCTGACCCAGAGTGGT

p21: cyclin-dependent kinase inhibitor 1A; BAX: BCL2 associated X, apoptosis regulator; GAPDH: glyceraldehyde-3-phosphate dehydrogenase.

### Flow cytometry

γ-H2AX expression was monitored in U2OS-shCTR and U2OS-shp53 cells after Eto treatment, after which they were incubated for the indicated time intervals and fixed in 70% ethanol overnight. The cells were permeabilized (0.25% Triton X-100 on ice for15 min), washed, incubated overnight in PBS containing 0.1% BSA and γ-H2AX antibodies, and then incubated with the secondary antibody for 30 min at room temperature. The cells were incubated in 50 µg/mL propidium iodide (PI) and 100 µg/mL RNase A for 30 min, and 1 × 10 ^4^ cells per sample were analyzed. For cell cycle analysis, U2OS cells were washed with PBS, detached by trypsin treatment, and collected by centrifugation at 200 *g* for 5 min. Then, the cells were washed with cold PBS and fixed in 70% ethanol for 24 h. The cells were then washed twice with cold PBS, resuspended in PBS with 50 μg/mL RNase A and incubated for 30 min at 37°C. The suspension was then added to PI solution (10 μg/mL) and incubated in the dark for 15 min. The relative DNA content of the stained cells was analyzed via flow cytometry. Flow cytometry was performed using a FACSCanto II (BD Biosciences, San Jose, USA), and the data were analyzed with FACSDiva software (BD Biosciences).

### Quantitative real-time polymerase chain reaction (qRT-PCR)

Total RNA was isolated from cultured cells via the TRIzol reagent, and the resulting messenger RNA (mRNA) was reverse transcribed into complementary DNA (cDNA) via the PrimeScript™ RT reagent Kit (TaKaRa) according to the manufacturer’s instructions. The qRT-PCR analysis was performed with SYBR ^®^ Premix Ex Taq™ (TaKaRa) following the manufacturer’s instructions. *GAPDH* and *β-actin* were used as internal controls. The 2 ^–ΔΔCt^ method was used to calculate the relative mRNA expressions of the target genes. The primers were designed according to the mRNA sequences of the target genes and reference genes in GenBank via Primer Premier 5 ( Table 2).

### Western blot analysis

Cells were lysed in RIPA buffer in the presence of PMSF and protease inhibitor cocktail (#7012; Cell Signaling Technology). Protein concentrations were quantified by using BCA protein assay reagent kit (Bioworld, Visalia, USA). Proteins were separated by 10% SDS-PAGE and transferred onto polyvinylidene difluoride (PVDF) membranes (Thermo Fisher Scientific). The membranes were blocked in 5% non-fat milk in Tris-buffered saline with 0.1% Tween 20 (TBST) for 1 h at room temperature (RT). After blocking, the membranes were incubated overnight at 4°C with specific primary antibodies diluted in blocking buffer. Then, the membranes were washed three times with TBST, followed by incubation with HRP-conjugated secondary antibodies for 1 h at RT. After washing with TBST, protein bands were visualized using SuperSignal ^TM^ West Pico PLUS (Thermo Fisher Scientific) and detected with the Amersham Imager 600 (GE Healthcare).

### Statistical analysis

Data were analyzed via Student’s *t* test and are presented as the mean ± SEM. The quantifications were based on the results of at least three independent experiments. The levels of significance are designated as *P* < 0.05.

## Results

### The expression level of p53 increases with the accumulation of DSB damage

**Figure 1:**
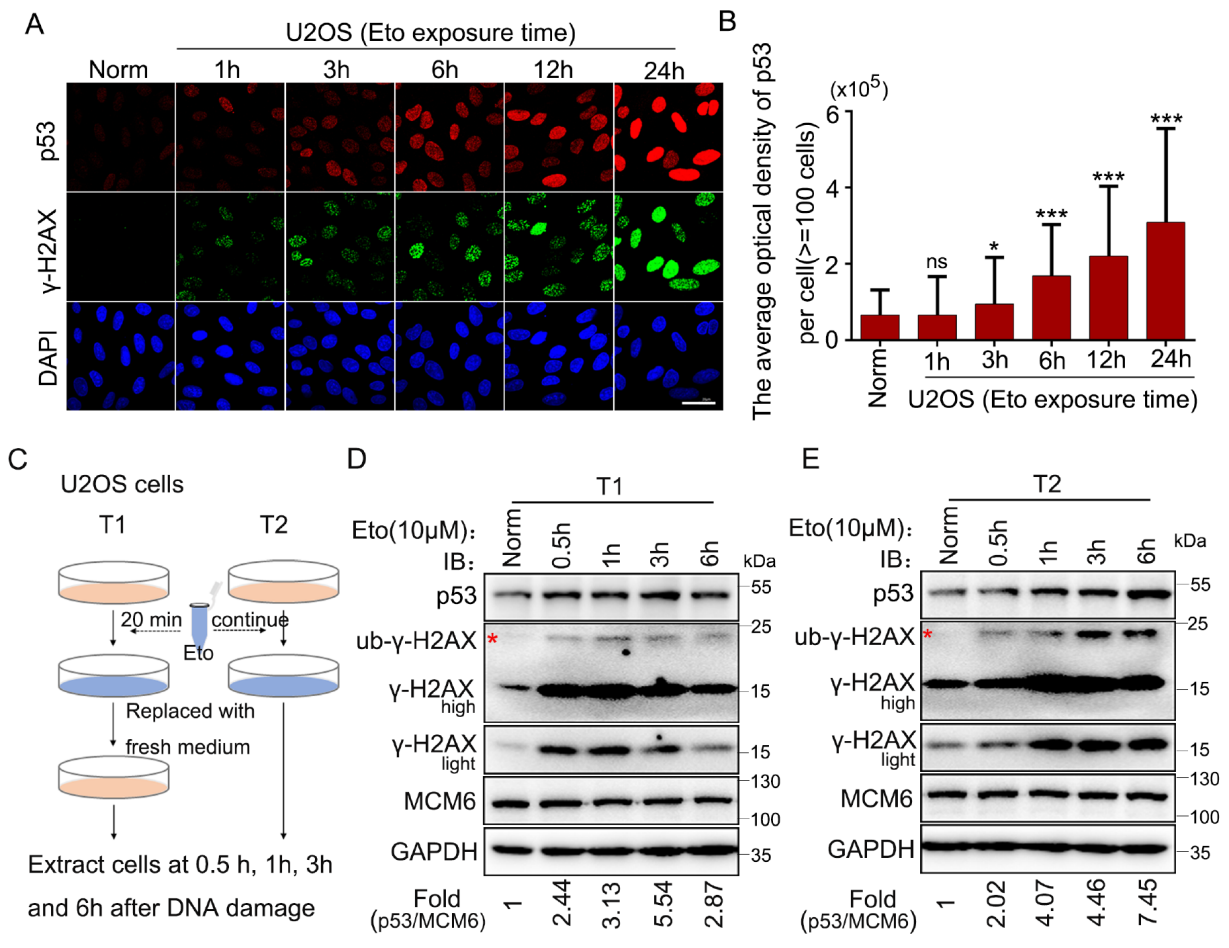
Figure 1 The expression level of p53 increased with increasing severity of DSB damage (A) U2OS cells were continuously stimulated with 10 μM Eto for the indicated time intervals. The cells were then fixed and immunostained with anti-p53 and anti-γ-H2AX antibodies. Scale bar: 40 μm. (B) Quantification of the average optical density of p53 protein per cell. n ≥ 3. *P < 0.05, ***P < 0.001. ns, not significant. (C) Schematic diagram illustrating the T1 and T2 treatment strategies. (D,E) U2OS cells were subjected to the T1 and T2 experimental methods, respectively, and the protein levels were analyzed via western blot analysis with the indicated antibodies. T1: HCT116 cells were exposed to 10 μM Eto for 20 min or not treated and then replaced by fresh medium and treated for the indicated time; T2: HCT116 cells were exposed to 10 μM Eto for the indicated time.

Etoposide (Eto) is an inhibitor of topoisomerase II. Immunofluorescence assays revealed that upon sustained exposure of U2OS cells to Eto, the level of the hallmark protein γ-H2AX, which is indicative of DSB damage, increased as the duration of drug exposure increased. Concurrently, the expression level of p53 also increased in line with the augmentation of DSB damage ( Figure 1A,B). These findings suggest that the increased expression of p53 could be positively correlated with the quantity of DSBs. To further validate whether the increased expression of p53 is indeed dependent on the severity of damage, we conducted confirmation experiments in U2OS cells. We designed two drug treatment strategies, T1 and T2 ( Figure 1C). In the T1 protocol, the cells were treated with Eto for 20 min, after which the medium was replaced by fresh culture medium. In contrast, the T2 protocol involves sustained exposure of the cells to the drug. The cells from both treatment conditions were then harvested at the designated time points for further western blot analysis. In the T1 protocol, following the induction of DSB damage and subsequent withdrawal of the drug, the level of γ-H2AX gradually increased, returning to baseline after 6 h, indicating that the damage repair process is incrementally accomplished. Similarly, the expression level of p53 also decreased at 6 h. However, in the T2 protocol, where there was continuous drug exposure, γ-H2AX remained highly expressed, and the expression level of p53 continued to accumulate. These results further indicate that p53 tends to accumulate persistently in response to an increase in DSB damage ( Figure 1D,E).

### p53 mediates DSB repair independent of the cell cycle and apoptosis

**Figure 2:**
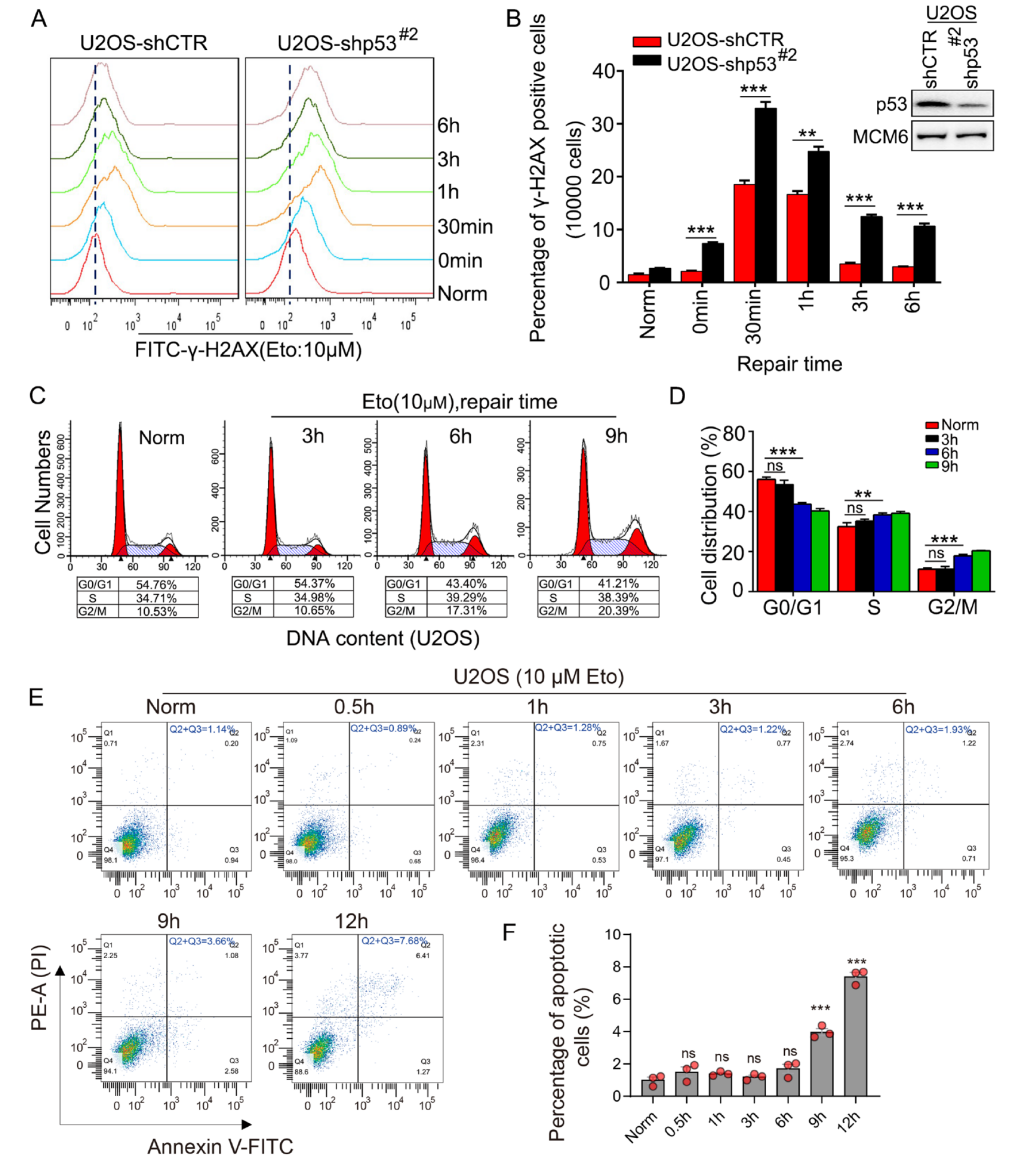
Figure 2 p53 directly mediates DSB repair independent of the cell cycle and apoptosis (A) U2OS cells stably transfected with shCTR or shp53 plasmids were treated with 10 μM Eto and allowed to repair for the indicated time. Flow cytometric analysis of γ-H2AX staining. A total of 1× 104 cells were analyzed in a single experiment, and the data shown are from a single representative experiment out of three replicates. (B) Quantification of γ-H2AX-positive cells. n ≥ 3. **P < 0.01, ***P < 0.001. The interference efficiency of the cells was detected via western blot analysis. (C) U2OS cells were treated with 10 μM Eto for 20 min or left untreated, after which the medium was replaced by fresh medium for the indicated time. The cell cycle distribution of U2OS cells was analyzed by flow cytometry. (D) The percentage of cells in each cell cycle phase is shown. n ≥ 3. **P < 0.01, ***P < 0.001. ns, not significant. (E) U2OS cells were treated with 10 μM Eto for the indicated time, and flow cytometry analysis of Annexin V-FITC/PI staining was conducted to examine the number of dead cells. (F) The percentage of total number of Annexin V-positive (Q3) and Annexin V/PI double-positive cells (Q2) was quantified. The P values were determined via an unpaired t test. ***P < 0.001. ns, not significant.

To further investigate whether p53 is involved in the regulation of DSB repair, we downregulated the expression of p53 in U2OS cells ( Supplementary Figure S1A). The different treatment groups were each exposed to Eto for 20 min, followed by replacement by fresh culture medium. The levels of γ-H2AX, a posttranslational modification of the H2AX protein indicative of DNA damage, were assessed at various time points via flow cytometry and western blot analysis. The results demonstrated that, compared with control treatment, *p53* knockdown led to significantly higher levels of γ-H2AX ( Figure 2A,B and Supplementary Figure S1B). These findings suggest that the knockdown of *p53* inhibits the efficiency of DSB repair.

Numerous studies have indicated that under DNA damage conditions, once p53 is translocated into the nucleus, it can maintain genomic stability either by inducing cell cycle arrest or promoting apoptosis [ 25, 31] . In our experiments, we stimulated cells with Eto for 20 min and subsequently replaced it by fresh culture medium to allow DSB repair. We examined the cell cycle distribution at various time points during the repair process and found no significant alterations within the first 3 h of repair. However, beyond 6 h, the cells began to arrest in the G2/M phase ( Figure 2C,D). We also assessed apoptosis in cells continuously treated with Eto for various durations and observed no apoptotic phenomena within 6 h ( Figure 2E,F). Additionally, we analyzed the mRNA expression levels of the classic p53 targets p21 and Bax and found that their expression levels did not significantly increase within 3 h ( Supplementary Figure S2). These findings suggest that during the early phase of DSB repair, the elevated expression of p53 does not primarily regulate cell cycle progression or apoptosis. These results indicate that p53 may directly participate in the process of DSB repair.

### p53 mainly participates in regulating the NHEJ repair pathway of DSB damage

**Figure 3:**
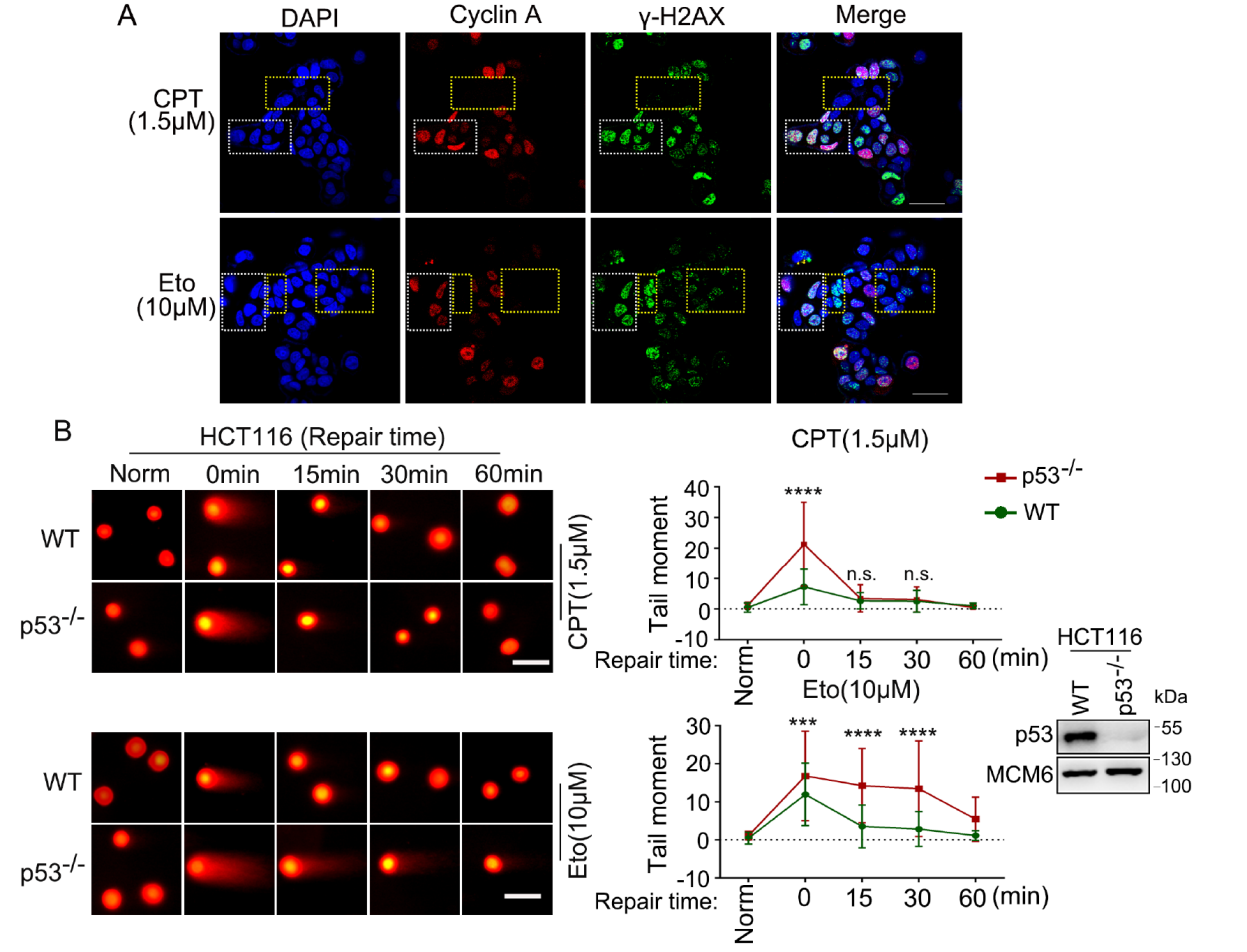
Figure 3 p53 affects mainly the NHEJ repair pathway of DSB damage (A) U2OS cells were treated with specific concentrations of CPT and Eto, followed by immunofluorescence staining of Cyclin A and γ-H2AX. (B) Neutral comet assay was performed on HCT116WT or HCT116p53–/– cells treated with 1.5 μM CPT or 10 μM Eto for 20 min, and the cells were collected at the indicated times. The right graph depicts the average tail moment of at least 50 cells; the error bars indicate the standard deviation (SD) values of three experiments. Comparisons were performed via Student’s t test. ***P < 0.001, ****P < 0.0001. ns, not significant.

Given that p53 may directly participate in the process of DSB repair and that DSB repair primarily occurs through the NHEJ and HR pathways, we are keen to determine which of these repair pathways is predominantly influenced by p53. Previous studies reported that DSB damage induced by camptothecin (CPT), an inhibitor of topoisomerase I, primarily relies on the HR repair pathway [32], whereas DSB damage caused by Eto predominantly depends on the NHEJ repair pathway [33]. Our findings in U2OS cells following treatment with both Eto and CPT are consistent with these reports [ 32, 33] . The damage induced by CPT mainly occurs in cells with high Cyclin A expression (Cyclin A is expressed primarily in the S and G2 phases), where HR repair also primarily occurs. Eto-induced DSB damage, on the other hand, was observed throughout the cell cycle, with an elevated incidence of damage in G1 phase cells ( Figure 3A). NHEJ repair can occur in all phases of the cell cycle. In light of the aforementioned characteristics, we employed CPT as a model to induce the HR repair pathway and utilized Eto as a model to stimulate the NHEJ repair pathway.

To explore the primary DSB repair pathways involving the p53 protein, we employed both *p53*-knockout (HCT116 ^p53–/–^) and wild-type p53 HCT116 (HCT116 ^WT^) cell lines. Evidence from comet assays revealed that, compared with that in wild-type cells, the absence of p53 results in an increased degree of DNA breakage, regardless of whether damage is induced by Eto or CPT. This effect was particularly pronounced at the 0-min mark, at the commencement of repair ( Figure 3B). However, the findings also indicated that under damage induced by CPT, the degree of DNA breakage in cells aligns with that in wild-type cells at the 15-min repair mark. In contrast, under damage induced by Eto, the extent of DNA breakage in p53-deficient cells remained significantly greater than that in wild-type cells within the first hour of repair. The above results suggest that at the early stages of DSB repair, the p53 protein not only rapidly stabilizes damage sites, preventing further DNA breakage but also enhances the efficiency of damage repair at breakage sites ( Figure 3B). This phenomenon is particularly apparent in the process of NHEJ repair induced by Eto, whereas its impact on HR repair processes induced by CPT is minimal. These results suggest that p53 primarily participates in regulating the early NHEJ repair pathway of DSB damage, especially within the first hour.

### p53 can be simultaneously recruited to both the proximal and distal sites of DSB damage

**Figure 4:**
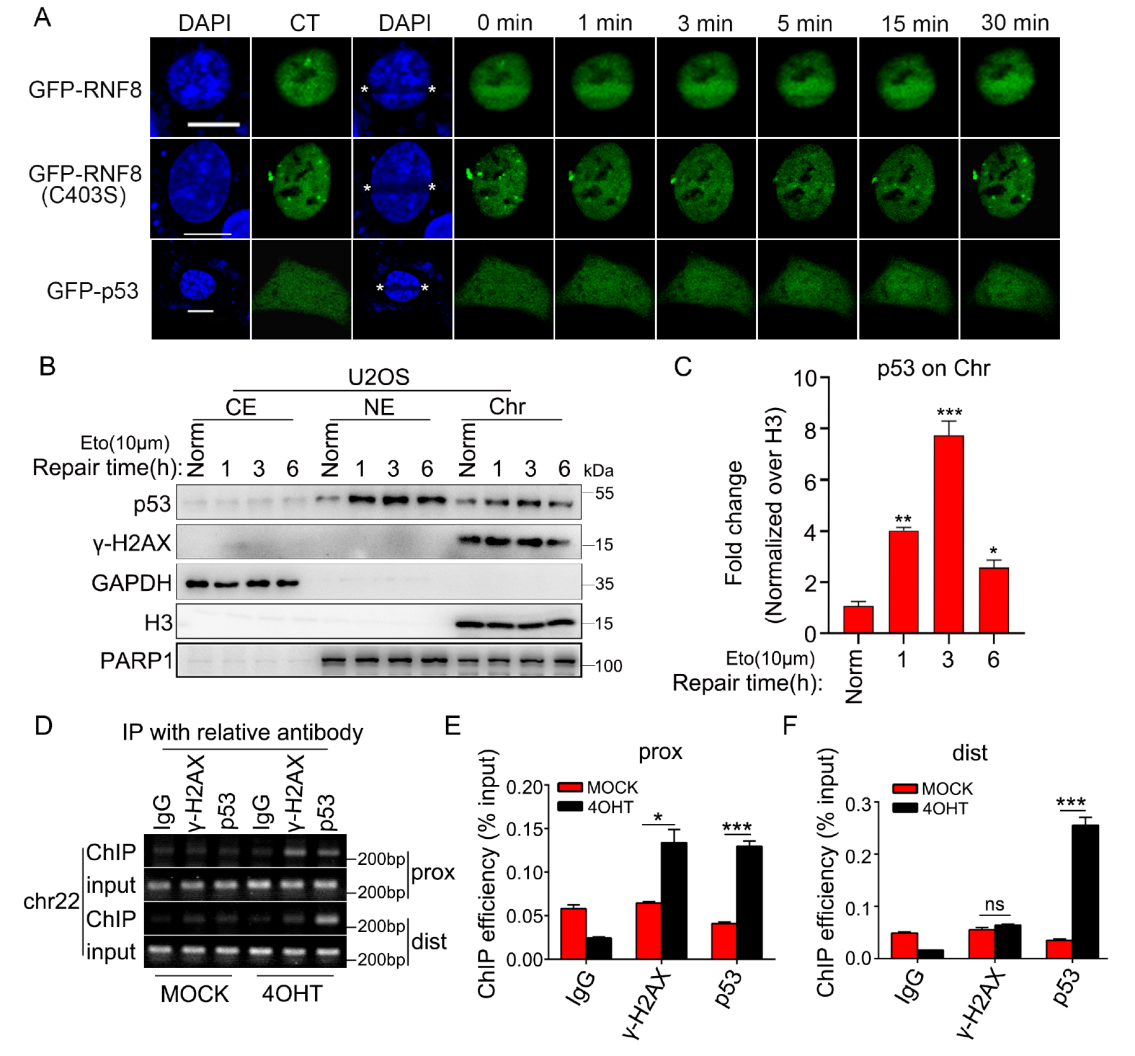
Figure 4 P53 can be simultaneously recruited to both the proximal and distal sites of DSB damage (A) U2OS cells in the exponential growth phase were transfected with GFP-tagged RNF8, its variant GFP-RNF8C403S, or GFP-p53 and subjected to treatment with Hoechst for 10 min. Subsequently, the cells were exposed to a focused laser microbeam that traversed linear tracks across the entire nuclear diameter. The cells were imaged at the indicated time points postexposure. (B) U2OS cells were initially treated with 10 μM Eto for 20 min, after which fresh culture medium was added for recovery. The cells were collected at the indicated time points. The cytoplasmic, nuclear, and chromatin fractions were then separated from the cells. The expression levels of p53 and γ-H2AX in different fractions were examined via western blot analysis. GAPDH, H3, and PARP1 were used as internal controls for the different fractions. (C) The corresponding statistical chart for the expression of p53 in the chromatin fraction (D) Chromatin immunoprecipitation (ChIP) was used to identify the binding of p53 and γ-H2AX at both the proximal and distal sites of DSB damage. (E,F) The corresponding statistical charts for (D). *P < 0.05, **P < 0.01, ***P < 0.001. ns, not significant.

We further investigated the mechanism by which p53 is involved in the repair of DSB damage. First, we induced DNA damage via UV-405 to ascertain whether p53 is directly recruited to the site of DSB damage. Previous studies revealed that the wild-type RNF8 protein can be recruited to the site of DSB damage, forming a repair focus, whereas the E3 ubiquitin ligase mutant RNF8 (RNF8 ^C403S^) cannot be recruited to the damage site [34]. In this context, we expressed the GFP-RNF8, GFP-RNF8 ^C403S^, and GFP-P53 proteins in U2OS cells. We then irradiated the cells with a laser to induce DNA damage and continuously monitored these proteins at the damage site. In line with previous reports, we found that RNF8 could be recruited to the damage site, whereas RNF8 ^C403S^ could not be recruited to the damage location ( Figure 4A). Compared with the control gene, p53 could not be significantly recruited to the DNA breakage site under conditions of DNA damage. However, the expression level of p53 throughout the cell nucleus increased ( Figure 4A). Additionally, the experiments separating different components of the cell nucleus, cytoplasm, and chromatin revealed that in the process of DSB repair, the accumulation of p53 in the cell nucleus and chromatin gradually increased, reaching a relatively high level at 3 h, and then gradually decreased over time ( Figure 4B,C). To further clarify the recruitment status of p53 to chromatin, we employed the Asi-ER-U2OS cell line used in previous studies [ 30, 35] . Through chromatin immunoprecipitation (ChIP) experiments, we found that p53 not only is recruited near the damage site but also significantly accumulates at distal sites of damage ( Figure 4D–F). We conclude that in the context of DSB damage, the accumulation of p53 in the cell nucleus gradually increases. It not only is recruited to DSB damage sites to exert its function but is also likely recruited across the surrounding chromatin, contributing to the maintenance of genome stability.

### p53 participates in regulating DSB repair by promoting chromatin structure relaxation

**Figure 5:**
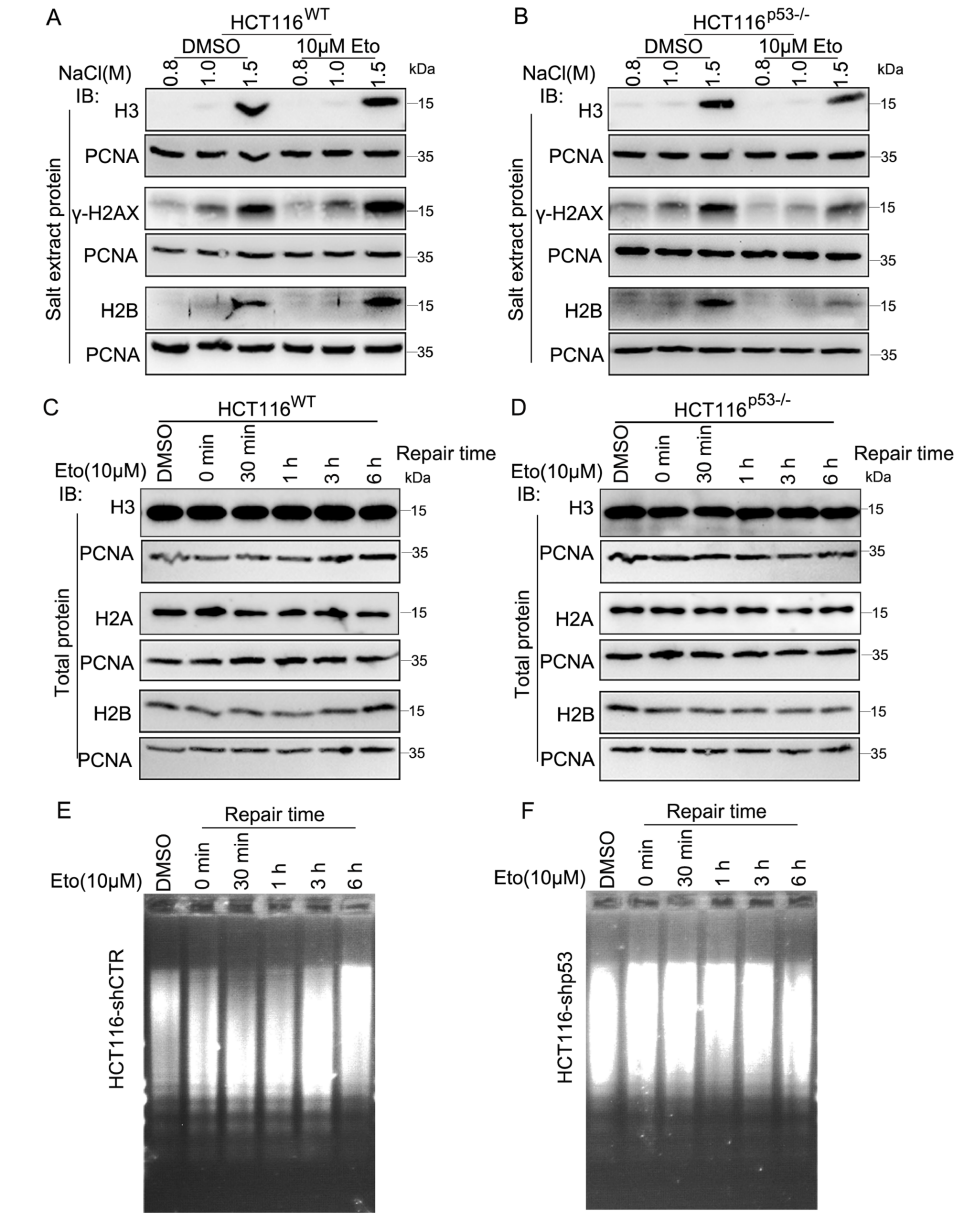
Figure 5 p53 participates in regulating DSB repair by promoting chromatin structure relaxation (A,B) HCT116WT and HCT116p53–/– cells were treated with DMSO or Eto for 20 min, followed by a recovery period of 30 min. The chromatin fractions of the cells were then extracted using different NaCl concentrations. The levels of the chromatin-associated proteins H3, γ-H2AX and H2B in the extraction supernatants were determined via western blot analysis. (C,D) HCT116WT and HCT116p53–/– cells were treated with DMSO or Eto for 20 min, followed by a recovery period of the indicated time. The overall protein levels of H3, γ-H2AX, and H2B were determined via western blot analysis. PCNA was used as an internal reference. (E,F) MNase accessibility of chromatin at different time points after treatment of HCT116WT and HCT116p53–/– cells with 10 μM Eto.

To further investigate the mechanism through which p53 is recruited to entire chromatin, we evaluated the overall open state of the chromatin. We conducted a sodium chloride (NaCl) extraction experiment on both wild-type and *p53*-knockout HCT116 cells. Typically, as the concentration of NaCl increases, more chromatin components are extracted. The results showed that in wild-type HCT116 cells, the quantity of chromatin components such as H3, γ-H2AX, and H2B extracted under the same concentration of NaCl was significantly greater in the Eto-treated group than in the untreated group ( Figure 5A). However, in *p53*-knockout HCT116 cells, the amount of chromatin components extracted following the same concentration of NaCl treatment in the Eto-treated group was actually lower than that in the untreated group ( Figure 5B). To rule out the possibility that the observed phenomena were due to a decrease in the overall protein levels, we evaluated the total protein expressions of H3, γ-H2AX, and H2B and found that there were no significant changes in the overall protein levels, regardless of whether the cells were in a wild-type state ( Figure 5C) or in a *p53*-knockout condition ( Figure 5D). In addition to the results in HCT116 cells, we also observed the same phenomenon in *p53*-knockdown U2OS cells ( Supplementary Figure S3). Moreover, the results from the chromatin micrococcal nuclease digestion experiment corroborated these findings. In cells with normal p53 expression, during damage repair, chromatin initially becomes progressively relaxed, and approximately 1 h after repair, it gradually condenses ( Figure 5E and Supplementary Figure S4A). Conversely, in cells with low p53 expression, the chromatin structure initially becomes denser during the DNA damage repair, peaks at approximately 1 h, and subsequently starts to gradually recover ( Figure 5F and Supplementary Figure S4B). These results indicated that p53 is capable of influencing the open state of chromatin during the DNA damage repair, thereby affecting the efficiency of DNA damage repair.

## Discussion

Given that genomic DNA is intricately interwoven within the highly organized chromatin structure, it is scientifically plausible that relaxation of the chromatin architecture is needed, thus permitting the necessary proteins to access and interact with damaged DNA segments [36]. Chromatin decompression has been extensively documented at sites of gene transcription, and several transcription activators have been shown to induce large-scale chromatin relaxation [37]. An enduring question in the DNA damage field has been whether chromatin undergoes decondensation following DNA damage. Evidence suggests that such process is indeed induced by DNA damage [38]. The tumor suppressor p53 has been reported to act as a chromatin accessibility factor, mediating UV-induced global chromatin relaxation for nucleotide excision repair (NER) [39]. However, whether the involvement of p53 in chromatin structure regulation to promote DNA damage repair is specific to the UV-induced NER pathway [39] or whether p53 has similar functions in other types of DNA damage repair is currently unclear.

Our work primarily investigated the regulatory function of p53 in the process of DSB repair. We found that the expression level of p53 is highly correlated with the severity of DSB damage. P53 can increase the efficiency of DSB repair by directly causing chromatin structure relaxation. The function of p53 occurs at the early stages of DSB repair, and it does not depend on p53-dependent cell cycle regulation or proapoptotic activity. In a compelling study, Yu-Hsiu Wang and colleagues [40] documented the impressive swift mobilization of p53 to sites of DNA damage induced by lasers. This intranuclear repositioning of p53 transpired with a median response time of merely 0.8 s subsequent to laser microirradiation. An in-depth examination of various domain-specific p53 mutants revealed that the swift accumulation of p53 does not necessarily correspond to the occurrence of transcriptional activity. This rapid aggregation of p53, which significantly influences the recruitment of 53BP1, preferentially triggers the activation of NHEJ repair. The implications of this process hold considerable relevance to our understanding of tumor suppression mechanisms [40]. In our studies of DSB damage induced by UV, etoposide, and the nuclease AsiSI, we found that at the early stages of DSB damage, p53 can be rapidly recruited to the site of damage and the surrounding chromatin. Knockdown of *p53* inhibited the efficiency of DSB repair. Comet assay experiments indicate that p53 mainly participates in the NHEJ pathway of DSB repair, with minimal impact on HR repair efficiency, which is consistent with the findings of previous studies [41]. Through chromatin component NaCl salt extraction experiments, we found that in cells where p53 is normally expressed, the chromatin structure rapidly loosens following DSB damage. We hypothesize that this may allow the damage recognition machinery to approach, identify, and localize damage sites. However, in cells in which *p53* is knocked out or expressed at low levels, the chromatin structure does not relax upon DSB damage but rather becomes even more compact than that in cells without DSB damage ( Figure 5). Under conditions where the overall expressions of chromatin-related components remain constant, the same concentration of NaCl can only extract a few histones. Regarding the mechanism by which p53 regulates the relaxation of chromatin structure at the early stages of DSB repair, we hypothesize that p53 has the biochemical capacity to induce chromatin relaxation, as it can mobilize the histone acetyltransferase (HAT) p300 toward chromatin, thereby facilitating histone acetylation—a critical step in the modification of chromatin structure [42]. In addition, recent work has revealed that specific p53 molecules bind to microsatellite sequences with limited similarity to the p53 consensus sequence, which may significantly increase the number of genomic p53-binding sites [43].

The expanding understanding of the nontranscriptional roles of p53 and its consequential contribution to tumor suppression has illuminated novel perspectives on how p53 orchestrates genome stability and thwarts tumorigenesis. Further in-depth investigations into the specific mechanisms by which p53 is rapidly recruited to damaged DNA and surrounding chromatin regions to promote chromatin relaxation under DSB damage conditions will provide additional theoretical groundwork for clinical cancer treatment.

## Supporting information

241FigS1-S4
