# Supplementary material for: p53-dependent chromatin relaxation is required for DNA double-strand break repair: p53 directly increases DSB damage repair efficiency
Source: Acta Biochim Biophys Sin (Shanghai). 2025 Feb 25;57(5):701–11. doi: 10.3724/abbs.2025008 (PMC12130726; doi:10.3724/abbs.2025008)
Supplement: 241FigS1-S4 [file 241FigS1-S4.docx]

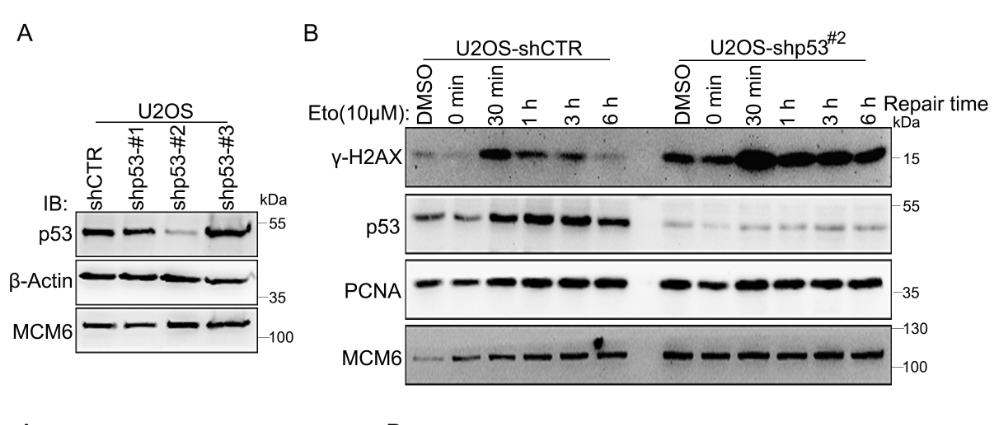


**Supplementary Figure S1.** **Knockdown efficiency of *p53* in U2OS cells and the impact of *p53* knockdown on DSB repair efficiency** (A) Western blot analysis was used to assess the knockdown efficiency of p53 protein. (B) U2OS cells stably transfected with shCTR or shp53 plasmids were treated with 10 μM of Eto and allowed to repair for the indicated time. Western blot analysis of the expression levels of γ-H2AX and p53. PCNA and MCM6 were used as internal references.


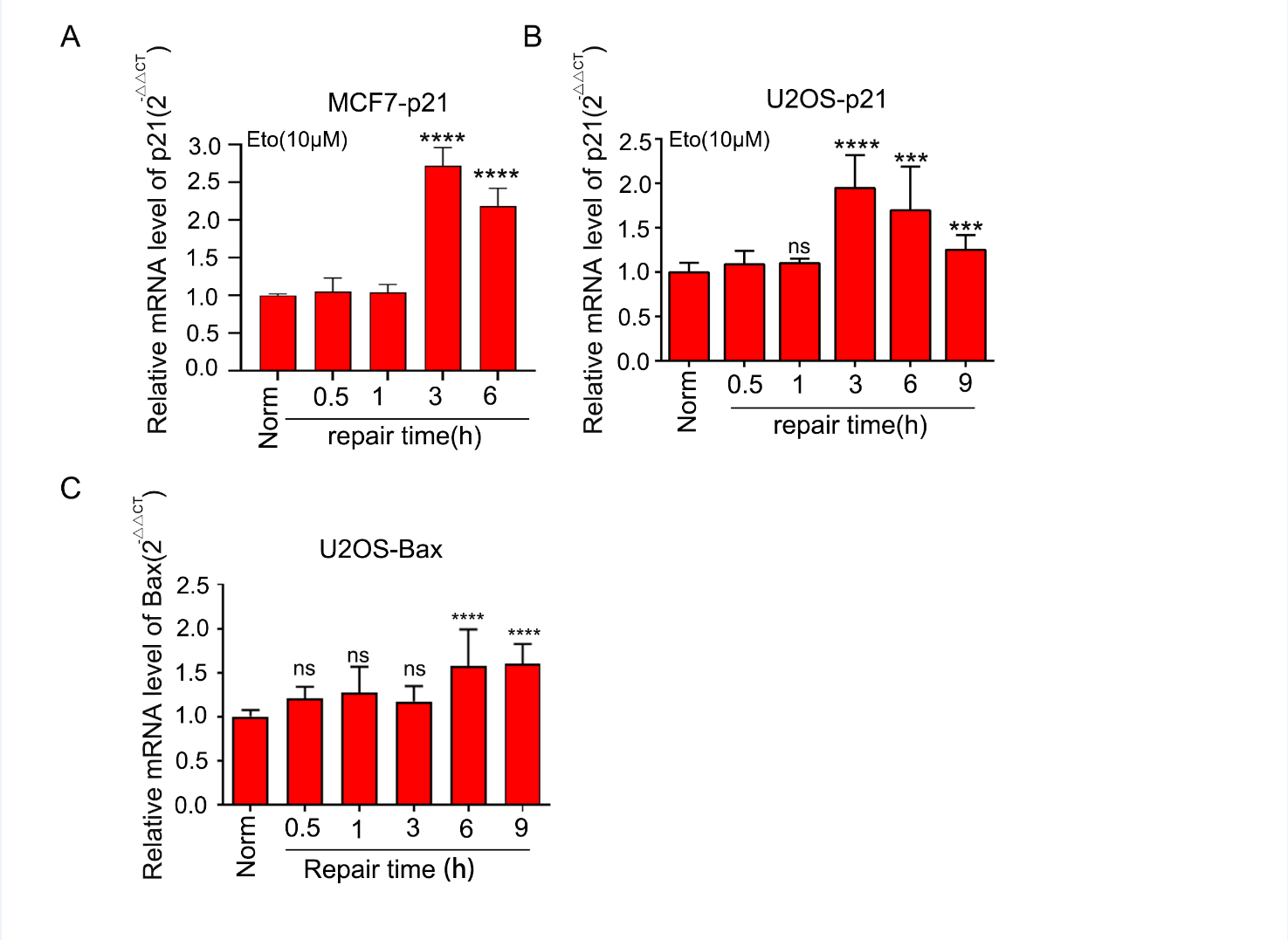


**Supplementary Figure S2.** **The relative mRNA levels of the indicated genes**  (A-C) The relative mRNA levels of *p21* and *Bax* were measured at various time points following Eto treatment to evaluate repair mechanisms in MCF7 and U2OS cells. The *P* values were determined using an ordinary one-way ANOVA multiple comparisons. ns, not significant; *** *P* < 0.001; **** *P* < 0.0001.


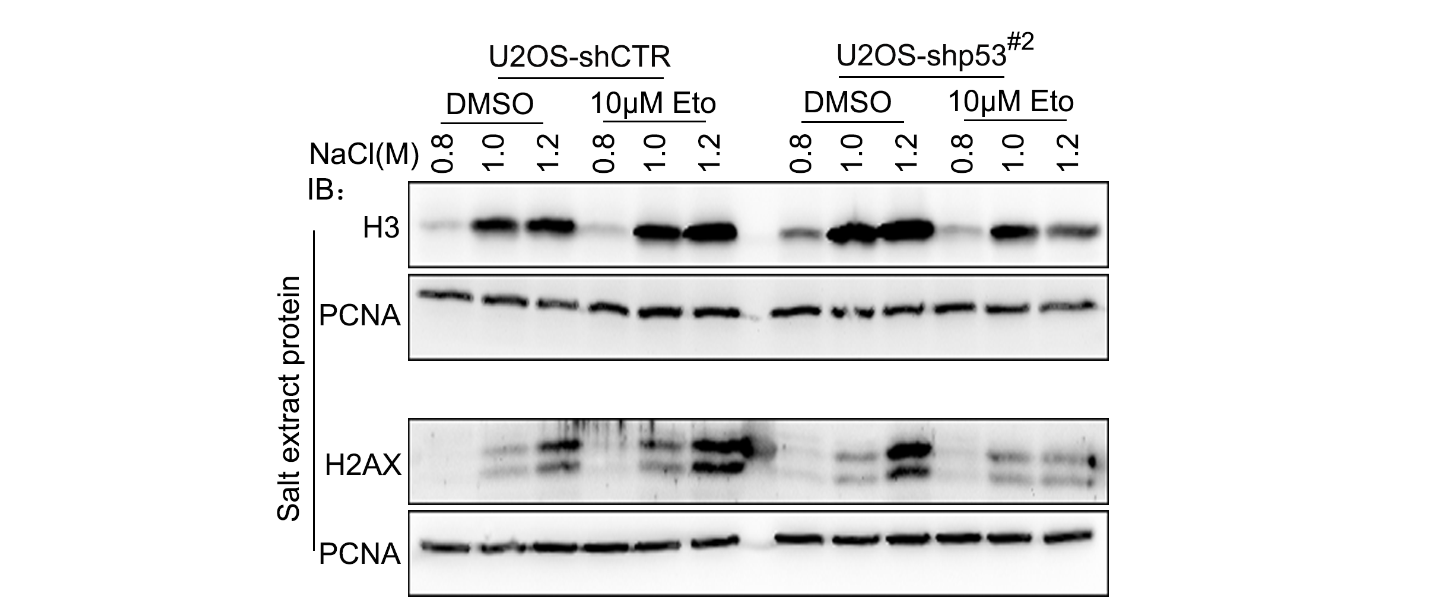


**Supplementary Figure S3** **Salt extraction experiments with different concentrations of NaCl were conducted in U2OS cells** U2OS-shCTR and U2OS-shp53^#2^ cells were respectively treated with DMSO and Eto for 20 min, followed by a recovery period of 30 min. The chromatin fractions of cells were then extracted using different NaCl concentrations. The levels of chromatin-associated proteins H3 and H2AX in the extraction supernatants were determined by western blot analysis.


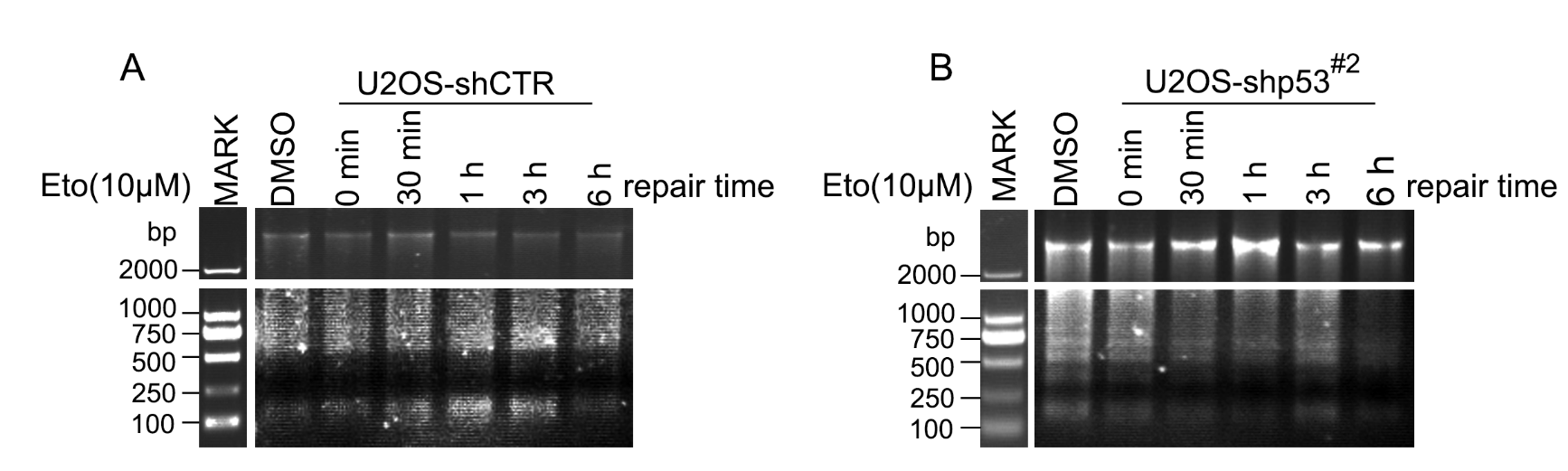


**Supplementary Figure S4** **Micrococcal nuclease assay was performed in U2OS cells**  The MNase accessibility of chromatin at different times after treatment of U2OS-shCTR and U2OS-shp53^#2^ cells with 10 μM Eto.
